# Genome-wide mapping of 5-hydroxymethyluracil in the eukaryote parasite *Leishmania*

**DOI:** 10.1186/s13059-017-1150-1

**Published:** 2017-01-30

**Authors:** Fumiko Kawasaki, Dario Beraldi, Robyn E. Hardisty, Gordon R. McInroy, Pieter van Delft, Shankar Balasubramanian

**Affiliations:** 1 Department of Chemistry, University of Cambridge, Lensfield Road, Cambridge, CB2 1EW UK; 2 Cancer Research UK Cambridge Institute, Li Ka Shing Centre, Robinson Way, Cambridge, CB2 0RE UK; 3 School of Clinical Medicine, University of Cambridge, Cambridge, CB2 0SP UK

**Keywords:** 5-Hydroxymethyluracil (5hmU), 5-Formyluracil (5fU), Base J, Genome-wide mapping, *Leishmania major*, *Leishmania donovani*

## Abstract

**Background:**

5-Hydroxymethyluracil (5hmU) is a thymine base modification found in the genomes of a diverse range of organisms. To explore the functional importance of 5hmU, we develop a method for the genome-wide mapping of 5hmU-modified loci based on a chemical tagging strategy for the hydroxymethyl group.

**Results:**

We apply the method to generate genome-wide maps of 5hmU in the parasitic protozoan *Leishmania* sp. In this genus, another thymine modification, 5-(β-glucopyranosyl) hydroxymethyluracil (base J), plays a key role during transcription. To elucidate the relationship between 5hmU and base J, we also map base J loci by introducing a chemical tagging strategy for the glucopyranoside residue.

Observed 5hmU peaks are highly consistent among technical replicates, confirming the robustness of the method. 5hmU is enriched in strand switch regions, telomeric regions, and intergenic regions. Over 90% of 5hmU-enriched loci overlapped with base J-enriched loci, which occurs mostly within strand switch regions. We also identify loci comprising 5hmU but not base J, which are enriched with motifs consisting of a stretch of thymine bases.

**Conclusions:**

By chemically detecting 5hmU we present a method to provide a genome-wide map of this modification, which will help address the emerging interest in the role of 5hmU. This method will also be applicable to other organisms bearing 5hmU.

**Electronic supplementary material:**

The online version of this article (doi:10.1186/s13059-017-1150-1) contains supplementary material, which is available to authorized users.

## Background

Natural chemical modifications to DNA nucleobases have the potential to profoundly influence biology. A number of modified bases have been identified in the genomes of a variety of organisms [1], such as derivatives of cytosine found in the genomes of mammals [2]. 5-Methylcytosine (5mC) and its oxidised derivatives 5-hydroxymethylcytosine (5hmC), 5-formylcytosine (5fC), and 5-carboxycytosine (5caC) have potential roles in gene regulation and epigenetics programming, which are fundamental in biology [3]. Yet, the details of thymine modifications and their roles in nature are still largely elusive. 5-Hydroxymethyluracil (5hmU) is a thymine base modification found in the genomic DNA of diverse organisms ranging from bacteriophages to mammals [1]. While the replicative incorporation of 5hmU is known in bacteriophage genomes [4, 5], the existence of enzyme-mediated pathways to form 5hmU in eukaryotic genomes suggests that this modification has functional importance [6]. The post-replicative formation of 5hmU occurs via hydroxylation of thymine, which can be mediated by the ten-eleven translocation (TET) enzymes and J-binding protein (JBP) family proteins in mammalian and protozoan genomes, respectively [6, 7]. 5hmU has also been reported to affect protein-binding to DNA [8] and may also be a key intermediate in the generation of site-specific mutations, as it can be excised by DNA glycosylases to create potentially mutagenic abasic sites [9]. As an important step towards understanding the functional importance of 5hmU, we have explored ways to detect and map 5hmU in genomic DNA. A method to map 5hmU:G base mispairs has been reported but will not be applicable to map 5hmU derived from thymine. [10] Although the single molecule, real-time (SMRT) sequencing approach could be an option [11], its application to map 5hmU in the actual genomic DNA has not been reported, possibly due to the relatively weak signals derived from 5hmU in SMRT sequencing [11] compared to the strong signature signals of nucleobases which have been sequenced by the approach successfully (e.g., 4mC, 6mA, and β-glucosylated modified bases) [12–15]. Here we report the first genome-wide map of 5hmU, which we determined for the parasitic protozoan *Leishmania* sp. using chemical manipulation of 5hmU and next-generation sequencing (NGS) [16]. In *Leishmania* sp., 5hmU is a key post-replication nucleobase modification that can be further modified enzymatically to form 5-(β-glucopyranosyl) hydroxymethyluracil (base J). mRNA genes in *Leishmania* sp. are transcribed as large continuous polycistronic gene clusters starting at small numbers of transcription initiation sites, accompanied by simultaneous post-transcriptional processing to produce mature mRNAs [17, 18]. Base J has been reported to play a major role in polycistronic transcription termination and has also been reported to localise at transcription initiation sites marked by histone acetylation, though any individual role of 5hmU has yet to be determined [19–21]. In this study, by individually mapping 5hmU and base J, we demonstrate genomic loci that are enriched exclusively with 5hmU and discuss potential unique consequences of 5hmU at these loci.

## Results

**Fig. 1:**
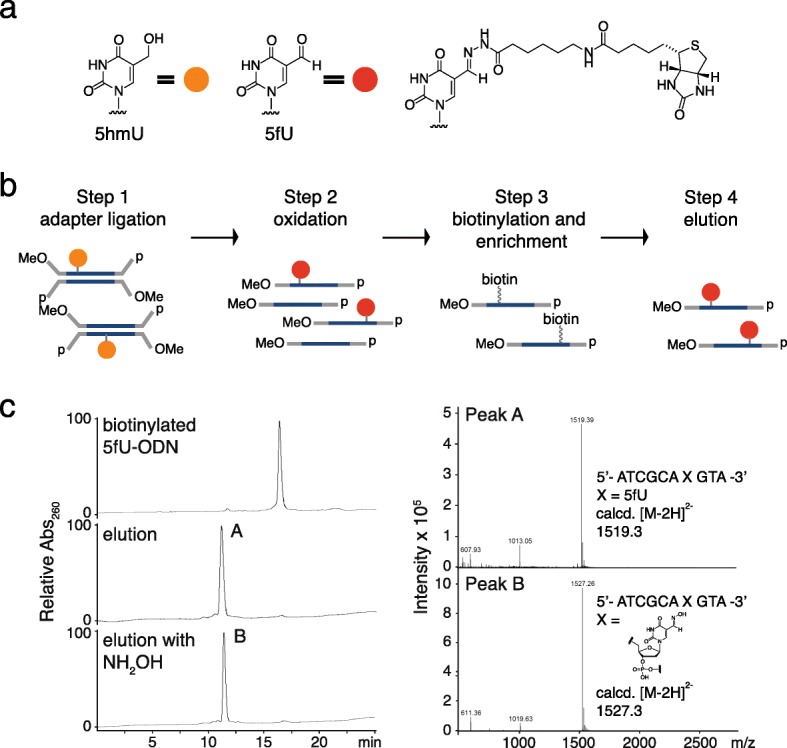
The 5hmU enrichment strategies. **a** 5-Hydroxymethyluridine (*5hmU*, *left*), 5-formyluridine (*5fU*, *middle*), and chemically tagged 5fU. **b** 5hmU chemical pull-down. *MeO* 5′-*O*-methyl, *p* 3′-phosphate. **c** LC-MS traces of 5fU-modified 10mer ODN (ODN-5fU) after biotinylation (*top*), followed by treatment in 20 mM phosphate buffer (pH 6) supplemented with *p*-anisidine (*center*) or 20 mM phosphate buffer (pH 6) supplemented with *p*-anisidine and NH_2_OH (*bottom*). Observed mass signals in peaks *A* and *B* (*right panel*)

The chemical enrichment of 5hmU-modified DNA fragments was carried out based on the selective chemical oxidation of 5hmU to 5-formyluracil (5fU) [16], followed by covalent biotinylation using an aldehyde-reactive probe (Fig. 1a, b). We first optimized the chemistry by tracing chemical reactions on a synthetic decamer oligodeoxynucleotide (ODN) model by liquid chromatography–mass spectrometry (LC-MS), which we further verified by a quantitative PCR enrichment study using 86mer ODNs (Fig. 1c; see Additional file 1: Tables S1 and S2 for the design of ODNs). We previously reported conversion of 5hmU to 5fU by KRuO_4_-mediated oxidation, by adapting the oxidation of 5hmC [16, 22]. To alleviate potential interference from oxidation of hydroxyl groups at both the 5′ and 3′ ends of each DNA fragment, we developed and utilized a custom NGS adapter bearing 5′-*O*-methyl groups and 3′-*O*-phosphate groups which render the DNA ends inert to the oxidation conditions. Biotinylation of 5fU-bearing DNA was achieved by reaction with (+)-biotinamideohexanoic acid hydrazide [16]. While the resulting covalent hydrazine linkage was stable in buffers used during the enrichment process, we could promote hydrolytic reversal of the linkage in slightly acidic media (pH 6), in the presence of the nucleophilic catalyst *p*-anidisine, to remove the biotin tag from DNA (Fig. 1c; Additional file 1: Figure S1a). Addition of hydroxylamine to the media further increased the recovery of DNA from magnetic beads by promoting removal of the hydrazone with the formation of a stable oxime (Fig. 1c; Additional file 1: Figure S1a). These findings provided a convenient method for elution of the enriched DNA from streptavidin beads following the pull-down while ensuring that eluted DNA fragments are free from substantial steric bulk introduced by the biotin group and linker moiety, which would otherwise decrease PCR efficiency [23].

We also assessed the reactivity of other aldehyde-bearing components that could potentially exist in genomic DNA, in particular abasic sites (APs) and 5fC, the latter of which can also derive from 5hmC during KRuO_4_-mediated oxidation. In the biotinylation condition we employed, only trace biotinylation of an AP-bearing ODN occurs whereas complete biotinylation of ODN-5fU was observed. Furthermore, the ODN was fragmented at the abasic site in the basic DNA denaturing condition followed by oxidization of 5hmU (Additional file 1: Figure S1b), so DNA fragments with abasic sites will not be subjected to sequencing since adapter sequences at both ends of the fragment are absolutely required. Taken together with the observation that AP-bearing DNA is also subjected to polymerase stalling during PCR (i.e., no amplification), [24] the effect of abasic sites in 5hmU enrichment would be negligible in the final sequenced data. Our DNA elution method would also reduce any background due to 5fC (which was absent in the *Leishmania* DNA we used in this study but present in mammalian genomes), as the biotin tag coupled to 5fU was removed faster and more selectively compared to the corresponding 5fC derivative in the optimum conditions (Additional file 1: Figure S1c). We then applied the enrichment chemistry to NGS library preparation (Step 3 in Fig. 1b). We also carried out the following controls in parallel (Additional file 1: Table S3): i) libraries prepared without the biotinylation and enrichment processes (i.e., “input” controls); ii) libraries prepared with the chemical pull-down without prior oxidation to control for peaks derived from any pre-existing modifications that react with the biotinylation probe as well as any biases caused by the enrichment process (i.e., “no-oxidation” controls).

**Fig. 2:**
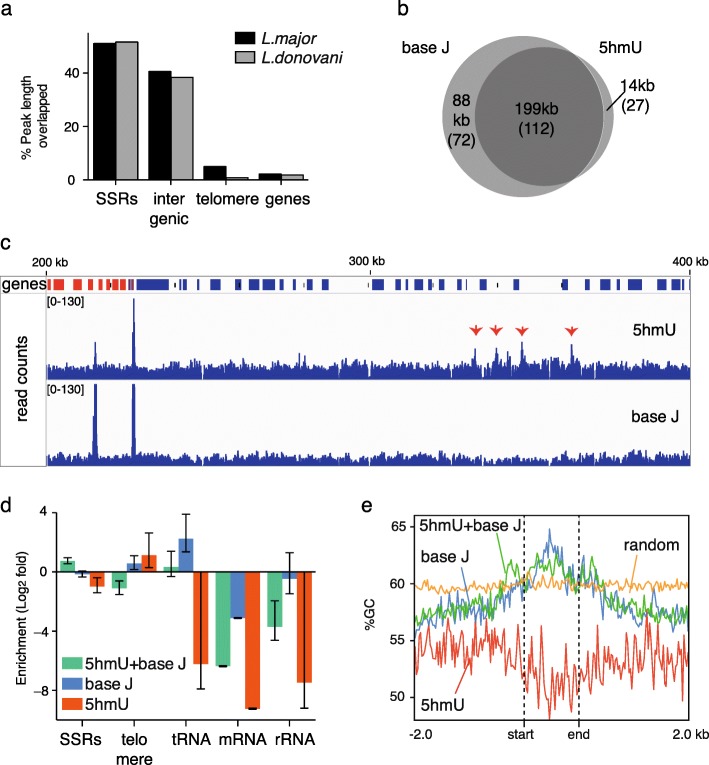
5hmU peaks in *L. major* obtained by DIP and chemical pull down. **a** Percentage of 5hmU peak lengths overlapped with genomic features. **b** Overlaps of 5hmU peaks and base J peaks in length (kb) and peak numbers (in *parentheses*). High confidence peaks were defined as peaks supported by at least two replicates out of three for each modification. Then the set of high confidence peaks were intersected to obtain consensus peaks for both 5hmU and base J. **c** An example of peaks in chromosome 23 (200–400 kb). 5hmU read counts (*top*) and base J read counts (*bottom*) with genes on the forward strand are shown in *red* and genes in the reverse strand are shown in *blue. Red arrows* indicate 5hmU loci. **d** Enrichment of peaks to each genomic feature. Values are shown with 95% confidence interval. **e** GC content (percentage) within peaks of T-modified loci and in the ±2kb flanking regions

We carried out genome-wide mapping of 5hmU in the eukaryotic parasites *Leishmania major* and *Leishmania donovani*. In these organisms, 5hmU levels were approximately 0.01% of all thymine bases by quantitative LC-MS (MS^2^)-based mononucleoside composition analysis (Additional file 1: Figure S2), which agrees with previous reports [25]. We regarded 5hmU peaks as high confidence when identified in at least two technical replicates out of three (151 5hmU peaks in *L. major* and 102 5hmU peaks in *L. donovani*). We also carried out 5hmU enrichment using DNA immunoprecipitation (5hmU-DIP) using a commercially available goat polyclonal IgG antibody which was found to bind specifically to 5-hmU bearing ODNs (Additional file 1: Figure S3a, b). We found, however, that the consistency of the peaks for the chemical enrichment was far greater than for the 5hmU-DIP (Additional file 1: Figure S3c), suggesting better robustness for the chemical 5hmU enrichment strategy. Only peaks obtained by the chemical enrichment were used for further analysis. 5hmU peaks were observed in the strand switch regions (SSRs; i.e., where the coding strand changes between large polycistronic gene clusters), telomeric regions, and intergenic regions (Fig. 2a). In both *L. major* and *L. donovani*, very few peaks were observed (up to four peaks) in no-oxidation controls. This observation suggests that the majority of 5hmU peaks we obtained are not a consequence of background signals, which might be attributed to pre-existing 5fU, or a potential minor reactivity of the probe to non-modified DNA (e.g., reactivity of amines to cytosine bases, as previously reported for hydroxyamines), [26] or non-specific binding of DNA to the magnetic resin.

**Fig. 3:**
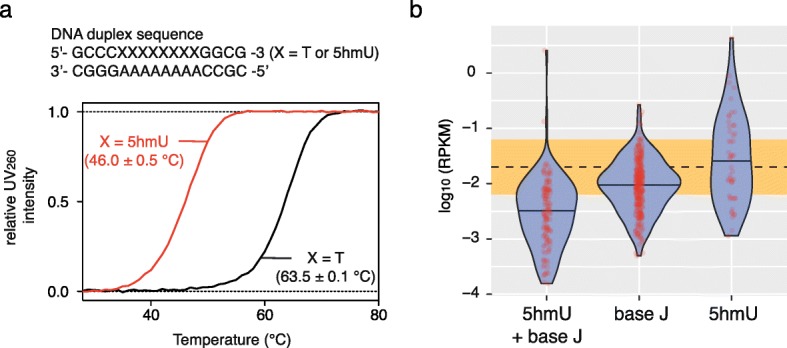
5hmU modification and related effects. **a** Thermal stability of DNA duplexes with oligo T (5hmU): oligo A contexts. Melting curves of a representative result and melting temperature values for each DNA duplex (mean ± standard deviation) are shown. **b** Violin plot showing normalized RNA expression as RPKM within 5hmU+J loci, 5hmU loci, and base J loci. The mean RNA count across random genomic regions (±1 standard deviation) is shown as the *orange bar*

To investigate the link between 5hmU loci and base J loci, we also established a chemical pull-down technique to enrich base J by adapting a chemical functionalization strategy for 5-(β-glucopyranosyl) hydroxymethylcytosine [27]. We applied this method to generate an enrichment-based map of base J in *L. major*, where the level of base J (approximately 0.08% of nucleobases) was higher than that of 5hmU (Additional file 1: Figure S2). Oxidation of the diol groups by NaIO_4_ on the glucopyranoside moiety of base J generates aldehydes, while 5hmU remained chemically inert (Additional file 1: Figure S4a). The resultant aldehyde groups were then biotinylated with (+)-biotinamideohexanoic acid hydrazide to allow the enrichment of base J-containing DNA fragments. The base J peaks obtained by our method agreed well (66% overlap in length) with the previously reported base J loci enriched by JBP, [20]; thus, we used the peaks to compare with 5hmU peaks (Additional file 1: Figure S4b). High confidence base J peaks (peaks supported by at least two technical replicates out of three) were observed for 93% of 5hmU-enriched loci (5hmU + base J loci) (Fig. 2b), consistent with the understanding that 5hmU is a precursor of base J [7]. Interestingly, we observed that some loci were uniquely detected by either 5hmU (5hmU-specific loci) or base J (base J-specific loci) enrichment (Fig 2c, d). While base J-specific loci were enriched over some genes, such as tRNA genes (Fig 2d), 5hmU-specific loci were mostly observed in intergenic regions, though no significant enrichment to any specific genomic feature was observed. It was found that 5hmU-specific loci had AT-rich contexts, whereas base J-specific loci and 5hmU + base J loci generally had GC-rich contexts (Fig. 2e). Motifs observed within 5hmU-specific loci by DREME [28] are characterized by a stretch of T, which was not observed for base-J specific loci (Additional file 1: Table S4). In a biophysics study using synthetic ODN duplex with an oligo T context, we observed that substitution of T by 5hmU decreased the thermal stability of the duplex (Fig. 3a), suggesting that 5hmU may also change the physical properties of local DNA duplex in the genome. To obtain insights into the 5hmU-specific loci, we analyzed the RNA levels in *L. major*. Levels of total RNA within thymine modification-enriched genomic loci were analyzed by counting RNA reads obtained from strand-directional total RNA sequencing. While RNA levels in 5hmU + base J loci and base J-specific loci were significantly lower than the average count (*p* < 0.001), they did not decrease in the 5hmU-specific loci, indicating that these loci remain transcriptionally active (Fig. 3b).

## Discussion

In the currently suggested mechanism of thymine modifications in trypanosomatids, the initial step of 5hmU formation is binding of the JBP family of proteins to base J, followed by hydroxymethylation of proximal thymine [25, 29]. The presence of base J-specific loci may suggest that any 5hmU formed is quickly enzymatically glycosylated to form base J in the loci. Although 5hmU-specific loci may have arisen from DNA oxidative damage regularly occurring in the genome, it is also possible that these loci form naturally via a JBP-independent mechanism. While previous studies have proposed that base J-associated glucosyltransferase in trypanosomatids readily converts 5hmU to base J promiscuously without sequence biases [15, 25, 30], the presence of 5hmU-specific loci suggests that some 5hmUs may exist in sequence contexts that are less preferred by this glucosyltransferase and are only slowly converted to base J. To assess the potential artifacts and biases caused by 5hmU chemical enrichment, especially due to spontaneous oxidation of T during library preparation, we carried out mock chemical 5hmU enrichment using a randomly sequenced ODN bearing no nucleoside modification, which will mimic the background signal. We did not observe any significant change in the nucleoside composition and enrichment of specific motifs, suggesting chemical 5hmU enrichment does not cause artifacts and biases (Additional file 1: Figure S5), which supports our interpretation of 5hmU-specific loci.

## Conclusions

We provide the first genome-wide map of 5hmU in *Leishmania sp.* genomes and investigated the relationship of 5hmU with the hypermodified base J. To achieve this goal, we established methods to selectively tag each modified derivative of the thymine base. The observed accumulation of 5hmU in T(A) rich sequence contexts, distinct from the sequence context of base J loci, suggests 5hmU remains intact or is less reactive to glucosylation in these sequence contexts. The methods presented herein provide powerful tools to help address the roles of modified T nucleobases, which are of emerging interest due to their relevance in biological function and disease [31–33].

## Methods

### Materials

The genomic DNA samples for *L. major* ATCC 30012D (lot 60685413, lot 63717803) as well as a set of genomic DNA and RNA from the same culture (lot ATCC-CUST-30012D/R) were obtained from ATCC*.* The genomic DNA sample for *L. donovani GR383WT1* (single batch) was provided courtesy of Prof. Charles L. Jaffe (National Center for Leishmaniasis, Kuvin Centre for Study of Tropical & Infectious Diseases, Jerusalem). Detailed information of samples used to generate each sequencing result is available in Additional file 1: Table S3.

### The modified adapter solution

A 5′-*O*-methylated ODN with a sequence of 5′-MeO-GAATGATACGGCGACCACCGAGATCTA CACTCTTTCCCTACACGACGCTCTTCCGATCT-3′ (Eurogentec) and a 3′-*O*-phosphorylated ODN with a sequence of 5′-GATCGGAAGAGCACACGTCTGAACTCCAGTCACNNNNNNATCTCGTATGCCGTCT TCTGCTTG-*O*-phosphate-3′ (Sigma Aldrich), where NNNNNN corresponds to TruSeq (Illumina) barcoding, were made up to a solution (25 μM each) in 50 mM NaCl and 20 mM Tris-HCl (pH 7) and annealed prior to use.

### Adapter ligation for chemical 5hmU enrichment

Fragmented DNA samples (200 ng) with an average size of 200 bp and spike-in control ODNs (100 pg each; see Additional file 1: Table S2) were mixed and subjected to adapter ligation using the standard protocol using the NEBNext® Ultra™ DNA Library Prep Kit for Illumina® (New England Biolabs), except the use of the modified adapter solution (2.5 μL) instead of a standard adapter solution. Adapter-ligated DNA samples were bound to AMPure® XP Beads (Beckman Coulter), washed two times with 80% aqueous acetonitrile (1.0 mL), eluted with 25 μL of water, then passed through Micro Bio-Spin™ P-6 Gel Columns in SSC Buffer (Bio-Rad) pre-washed five times with ultrapure water (0.5 mL). It was essential to avoid use of ethanol during the DNA sample purification.

### Chemical modifications of 5hmU

The adapter ligated DNA sample in 50 mM NaOH (24 μL) was denatured by incubating at 37 °C for 30 min. A ten-fold diluted oxidant solution (1 μL) provided in the TrueMethyl™ kit (Cambridge Epigenetics) was added to the DNA solution and the resultant mixture was incubated at 40 °C for 30 min, followed by immediate purification by Micro Bio-Spin™ P-6 Gel Columns (Bio-Rad). The resultant DNA sample (in ca. 25 μL solution), 100 mM phosphate buffer (pH 6, 10 μL), and 17 mM (+)-biotinamidohexanoic acid hydrazide (15 μL) were mixed and incubated at 40 °C for 3–12 h. The reaction mixture was purified by Micro Bio-Spin™ P-6 Gel Columns (Bio-Rad) and used immediately for enrichment.

### Chemical 5hmU enrichment sequencing

The biotinylated DNA sample (in ca. 50 μL solution) and the binding buffer 1 (2×, 50 μL; see Additional file 1 for the buffer composition) were mixed and added to Dynabeads® MyOne™ Streptavidin C1 (Life Technologies) which had been pre-washed two times with the binding buffer 1 (200 μL). The mixture was gently mixed by rotation at 4 °C for 30 min, placed on a magnetic rack, and the supernatant was removed. The beads were washed five times with the binding buffer 1 (1×, 100 μL). The DNA was eluted by incubating the beads in elution buffer (1×, 50 μL; see Additional file 1 for the buffer composition) at 40 °C with vigorous shaking (1400 rpm) for 30 min. The mixture was then placed on a magnetic rack and the supernatant containing the eluted DNA was collected. The elution process was repeated once more. The combined eluted DNA solutions were purified using the GeneJET PCR purification kit (Thermo Scientific). The obtained 5hmU enriched libraries were amplified by PCR Master Mix and PCR Primer Cocktail provided in the TruSeq DNA Sample Preparation Kit v3 (Illumina) and quantified using a KAPA Library Quantification Kit (KAPA Biosystems). Experiments were carried out in technical triplicates for *L. major* and technical duplicates for *L. donovani*. The efficacy of 5hmU enrichment was confirmed by read counts for 5hmU-modified spike-in controls over non-modified controls (Additional file 1: Table S3).

### Chemical base J enrichment sequencing

A solution of the fragmented DNA samples (250 ng) with an average length of 200 bp, 50 mM NaIO_4_ in 50 mM sodium acetate buffer (pH 5.5) was incubated at 40 °C for 3 h followed by work-up with Micro Bio-Spin™ P-6 Gel Columns (Bio Rad) or a GeneJET PCR purification kit (Thermo Scientific). The resultant DNA solution (25 μL), 100 mM phosphate buffer (pH 6, 20 μL), 100 mM (+)-biotinamidohexanoic acid hydrazide (4 μL), and 100 mM *p*-anisidine (1 μL) were mixed and incubated at 40 °C for 4–12 h. The reaction mixture was purified by Micro Bio-Spin™ P-6 Gel Columns (Bio Rad) or a GeneJET PCR purification kit (Thermo Scientific). The samples were then subjected to NGS adapter ligation using reagents provided in the NEBNext® Ultra™ DNA Library Prep Kit for Illumina® (New England Biolabs) and adapter solutions provided in the TruSeq DNA Sample Preparation Kit v3 (Illumina). Downstream procedures for base J enrichment were carried out as described in the chemical 5hmU pull-down sequencing procedure except for the use of KAPA HiFi HotStart Uracil + ReadyMix (KAPA Biosystems) as a PCR master mix.

### Sequence alignment and peak calling

5hmU and base J enriched libraries as well as control libraries were sequenced on an Illumina MiSeq with a read length of 151 bp. Reads were trimmed to remove adapters and low quality 3′ ends using cutadapt version 1.7 [34]. Trimmed reads were then aligned to the reference genome using bwa mem version 0.7.10 [35]. Only primary alignments with a mapping quality of 10 or more were retained for further analyses. Alignment filtering and manipulations were performed with samtools version 1.1 [35]. See Additional file 1: Table S3 for a summary of the mapping step. Peaks of read enrichment were detected using macs [36] version 2.1.0.20140616. Further analyses were performed by means of bedtools [37], deepTools [38], DREME [28], and custom bash, R, and python scripts.

## Additional files

Additional file 1: The datasets supporting the conclusions of this article as well as experimental designs and protocols are included within the article and its additional file. **Table S1.** Short ODNs used for the LC-MS study. **Table S2.** Sequences of ODN templates and primers used to synthesize model ODNs by PCR. **Figure S1.** Chemical pull-down of modified ODNs. **Figure S2.** DNA nucleobase modification profiles of *L. major* and *L. donovani* GR383WT1. **Figure S3.** Binding specificity of the antibody (ab19735) used for 5hmdU-DIP. **Figure S4.** Base J chemical pull down. **Figure S5.** Assessment of experimental biases. **Table S3**. Libraries prepared in this study. **Table S4.** DREME motif sequences and additional experimental details for synthesis of oligo DNA models, preparation of DNA models, reaction with oligo DNA models, quantitative PCR analysis to assess enrichment efficacy of chemical pull-down, affinity validation of ab19735 by ELISA, mononucleotide composition analysis, 5hmU-DIP, melting temperature analysis, and total RNA sequencing. (PDF 1440 kb)

## Abbreviations

5fC: 5-Formylcytosine
5fU: 5-formyluracil
5hmC: 5-Hydroxymethylcytosine
5hmU: 5-Hydroxymethyluracil
base J: 5-(β-glucopyranosyl) hydroxymethyluracil
DIP: DNA Immunoprecipitation
JBP: J-binding protein
LC-MS: Liquid chromatography–mass spectrometry
NGS: Next-generation sequencing
ODN: Oligodeoxynucleotide
SSR: Strand switch region

## References

[1] Gommers-Ampt JH, Borst P (1995). Hypermodified bases in DNA. FASEB J.

[2] Fu Y, He C (2012). Nucleic acid modifications with epigenetic significance. Curr Opin Chem Biol.

[3] Teif VB, Rippe K (2015). Interplay of nucleosome repositioning, DNA methylation and transcription factor binding during stem cell development. J Biomol Struct Dyn.

[4] Neuhard J, Maltman KL, Warren RA (1980). Bacteriophage phi W-14-infected Pseudomonas acidovorans synthesizes hydroxymethyldeoxyuridine triphosphate. J Virol.

[5] Witmer H (1981). Synthesis of deoxythymidylate and the unusual deoxynucleotide in mature DNA of Bacillus subtilis bacteriophage-Sp10 occurs by post-replicational modification of 5-hydroxymethyldeoxyuridylate. J Virol.

[6] Pfaffeneder T, Spada F, Wagner M, Brandmayr C, Laube SK, Eisen D, Truss M, Steinbacher J, Hackner B, Kotljarova O (2014). Tet oxidizes thymine to 5-hydroxymethyluracil in mouse embryonic stem cell DNA. Nat Chem Biol.

[7] Borst P, Sabatini R, Base J (2008). Discovery, biosynthesis, and possible functions. Annu Rev Microbiol.

[8] Greene JR, Morrissey LM, Foster LM, Geiduschek EP (1986). DNA binding by the bacteriophage SPO1-encoded type II DNA-binding protein, transcription factor 1. Formation of nested complexes at a selective binding site. J Biol Chem.

[9] Jacobs AL, Schar P (2012). DNA glycosylases: in DNA repair and beyond. Chromosoma.

[10] Yu M, Song CX, He C (2015). Detection of mismatched 5-hydroxymethyluracil in DNA by selective chemical labeling. Methods.

[11] Clark TA, Spittle KE, Turner SW, Korlach J (2011). Direct detection and sequencing of damaged DNA bases. Genome Integr.

[12] Song CX, Clark TA, Lu XY, Kislyuk A, Dai Q, Turner SW, He C, Korlach J (2011). Sensitive and specific single-molecule sequencing of 5-hydroxymethylcytosine. Nat Methods.

[13] Korlach J, Turner SW (2012). Going beyond five bases in DNA sequencing. Curr Opin Struct Biol.

[14] van Luenen H, Genest PA, Zhao W, Jan S, Baugh L, Clark T, Turner S, Korlach J, Myler P, Borst P (2014). SMRT sequencing defines the sequence requirements for the positioning of base J into DNA. FEBS J.

[15] Genest PA, Baugh L, Taipale A, Zhao WQ, Jan S, van Luenen HGAM, Korlach J, Clark T, Luong K, Boitano M (2015). Defining the sequence requirements for the positioning of base J in DNA using SMRT sequencing. Nucleic Acids Res.

[16] Hardisty RE, Kawsaki F, Sahakyan AB, Balasubramanian S (2015). Selective chemical labeling of natural T modifications in DNA. J Am Chem Soc.

[17] Ivens AC, Peacock CS, Worthey EA, Murphy L, Aggarwal G, Berriman M, Sisk E, Rajandream MA, Adlem E, Aert R (2005). The genome of the kinetoplastid parasite, Leishmania major. Science.

[18] Clayton C, Shapira M (2007). Post-transcriptional regulation of gene expression in trypanosomes and leishmanias. Mol Biochem Parasitol.

[19] Thomas S, Green A, Sturm NR, Campbell DA, Myler PJ (2009). Histone acetylations mark origins of polycistronic transcription in Leishmania major. BMC Genomics.

[20] van Luenen HG, Farris C, Jan S, Genest PA, Tripathi P, Velds A, Kerkhoven RM, Nieuwland M, Haydock A, Ramasamy G (2012). Glucosylated hydroxymethyluracil, DNA base J, prevents transcriptional readthrough in Leishmania. Cell.

[21] Reynolds D, Cliffe L, Forstner KU, Hon CC, Siegel TN, Sabatini R (2014). Regulation of transcription termination by glucosylated hydroxymethyluracil, base J, in Leishmania major and Trypanosoma brucei. Nucleic Acids Res.

[22] Booth MJ, Branco MR, Ficz G, Oxley D, Krueger F, Reik W, Balasubramanian S (2012). Quantitative sequencing of 5-methylcytosine and 5-hydroxymethylcytosine at single-base resolution. Science.

[23] McInroy GR, Raiber EA, Balasubramanian S (2014). Chemical biology of genomic DNA: minimizing PCR bias. Chem Commun (Camb).

[24] Gruz P, Shimizu M, Pisani FM, De Felice M, Kanke Y, Nohmi T (2003). Processing of DNA lesions by archaeal DNA polymerases from Sulfolobus solfataricus. Nucleic Acids Res.

[25] Bullard W, da Rosa-Spiegler JL, Liu S, Wang YS, Sabatini R (2014). Identification of the glucosyltransferase that converts hydroxymethyluracil to base J in the trypanosomatid genome. J Biol Chem.

[26] Munzel M, Lercher L, Muller M, Carell T (2010). Chemical discrimination between dC and (5Me)dC via their hydroxylamine adducts. Nucleic Acids Res.

[27] Pastor WA, Pape UJ, Huang Y, Henderson HR, Lister R, Ko M, McLoughlin EM, Brudno Y, Mahapatra S, Kapranov P (2011). Genome-wide mapping of 5-hydroxymethylcytosine in embryonic stem cells. Nature.

[28] Bailey TL (2011). DREME: motif discovery in transcription factor ChIP-seq data. Bioinformatics.

[29] Cliffe LJ, Hirsch G, Wang J, Ekanayake D, Bullard W, Hu M, Wang Y, Sabatini R (2012). JBP1 and JBP2 proteins are Fe2+/2-oxoglutarate-dependent dioxygenases regulating hydroxylation of thymidine residues in trypanosome DNA. J Biol Chem.

[30] van Leeuwen F, Kieft R, Cross M, Borst P (1998). Biosynthesis and function of the modified DNA base beta-D-glucosyl-hydroxymethyluracil in Trypanosoma brucei. Mol Cell Biol.

[31] Djuric Z, Heilbrun LK, Lababidi S, Berzinkas E, Simon MS, Kosir MA (2001). Levels of 5-hydroxymethyl-2 ′-deoxyuridine in DNA from blood of women scheduled for breast biopsy. Cancer Epidemiol Biomark Prev.

[32] Frenkel K, Karkoszka J, Glassman T, Dubin N, Toniolo P, Taioli E, Mooney LA, Kato I (1998). Serum auto antibodies recognizing 5-hydroxymethyl-2 ′-deoxyuridine, an oxidized DNA base, as biomarkers of cancer risk in women. Cancer Epidemiol Biomark Prev.

[33] Ganguly T, Duker NJ (1992). Reduced 5-hydroxymethyluracil-DNA glycosylase activity in Werner’s syndrome cells. Mutat Res.

[34] Martin M. Cutadapt removes adapter sequences from high-throughput sequencing reads. EMBnetjournal. 2011;17. http://dx.doi.org/10.14806/ej.17.1.200.

[35] Li H. Aligning sequence reads, clone sequences and assembly contigs with BWA-MEM. 2013. arXiv:1303.3997.

[36] Zhang Y, Liu T, Meyer CA, Eeckhoute J, Johnson DS, Bernstein BE, Nusbaum C, Myers RM, Brown M, Li W, Liu XS (2008). Model-based analysis of ChIP-Seq (MACS). Genome Biol.

[37] Quinlan AR, Hall IM (2010). BEDTools: a flexible suite of utilities for comparing genomic features. Bioinformatics.

[38] Ramirez F, Dundar F, Diehl S, Gruning BA, Manke T (2014). deepTools: a flexible platform for exploring deep-sequencing data. Nucleic Acids Res.

