# Supplementary material for: Genome-wide mapping of 5-hydroxymethyluracil in the eukaryote parasite Leishmania
Source: Genome Biol. 2017 Jan 30;18:23. doi: 10.1186/s13059-017-1150-1 (PMC5282726; doi:10.1186/s13059-017-1150-1)
Supplement: Additional file 1: — The datasets supporting the conclusions of this article as well as experimental designs and protocols are included within the article and its additional file. Table S1. Short ODNs used for the LC-MS study. Table S2. Sequences of ODN templates and primers used to synthesize model ODNs by PCR. Figure S1. Chemical pull-down of modified ODNs. Figure S2. DNA nucleobase modification profiles of L. major and L. donovani GR383WT1. Figure S3. Binding specificity of the antibody (ab19735) used for 5hmdU-DIP. Figure S4. Base J chemical pull down. Figure S5. Assessment of experimental biases. Table S3. Libraries prepared in this study. Table S4. DREME motif sequences and additional experimental details for synthesis of oligo DNA models, preparation of DNA models, reaction with oligo DNA models, quantitative PCR analysis to assess enrichment efficacy of chemical pull-down, affinity validation of ab19735 by ELISA, mononucleotide composition analysis, 5hmU-DIP, melting temperature analysis, and total RNA sequencing. (PDF 1440 kb) [file 13059_2017_1150_MOESM1_ESM.pdf]

Supplementary Information

Genome-wide mapping of 5-hydroxymethyluracil in the eukaryote parasite Leishmania

Fumiko Kawasaki,[1] Dario Beraldi,[1,2] Robyn E. Hardisty,[1] Gordon R. McInroy,[1] Pieter van Delft,[1] and Shankar Balasubramanian[1,2,3*]

1 Department of Chemistry, University of Cambridge, Lensfield Road, Cambridge CB2 1EW, U.K.

2 Cancer Research UK Cambridge Institute, Li Ka Shing Centre, Robinson Way, Cambridge CB2 0RE, U.K.

3 School of Clinical Medicine, University of Cambridge, Cambridge CB2 0SP, U.K.

Table of contents

1. Supplementary figures and tables

    Table S1. Short ODNs used for LC-MS study

    Table S2. Sequences of ODN templates and primers used to synthesize model ODNs by PCR

    Fig. S1. Chemical pull down of modified ODNs

    Fig. S2. DNA nucleobase modification profiles of L. major and L. donovani GR383WT1

    Fig. S3. 5hmU specificity of the antibody used for 5hmU-DIP.

    Fig. S4. Base J chemical pull down

    Fig. S5. Assessment of experimental biases

    Table S3. Motif sequences obtained by DREME analysis

    Table S4. Libraries prepared in this study

2. Additional experimental details

    2-1. General information

    2-2. Preparation of DNA models listed in Table 2

    2-3. Reaction with oligo DNA models

    2-4. q-PCR analysis to assess enrichment efficacy of chemical pull down

    2-5. Validation of antibody ab19735 by ELISA

    2-6. Mononucleotide composition analysis

    2-7. 5hmU DIP-seq

    2-8. Melting temperature analysis

    2-9. Total RNA sequencing

1. Supplemental figures and tables

Table S1 | Short ODNs used for LC-MS study

ODN-5hmU[a]	5'-ATCGCAHGTA-3'	H = 5hmU
ODN-5fU[b]	5'-ATCGCAHGTA-3'	H = 5fU
ODN-5fC[a]	5'-TAATTATCTCHGGACTCATAAG-3'	H = 5fC
ODN-AP[c]	5'-CAGATHTACGATT-3'	H = abasic site
ODN-mix[b]	5'-GCAGH^1AH^2AH^3AGCG-3'	H^1 = 5fU, H^2 =5hmU, H^3 = base J

a) ODN-5hmU and ODN-5fC were obtained from commercial sources (ATD Bio and Eurogentec, respectively); b) ODN-5fU and ODN-mix were synthesized in procedures described elsewhere [1]; c) ODN-AP was prepared from uracil modified ODN (5'-CAGATUTACGATT-3', Sigma Aldrich) by treatment with 5U Uracil DNA Glycosylase (NEB) for 3h at 37 ºC followed by purification by mini quick-spin oligo column (Roche)

Table S2 | Sequences of ODN templates and primers used to synthesize model ODNs by PCR

ODN1[a]	
Template (5' to 3')	ATCGAGAATCCCGGTGCCGAGGCCGCTCAATTGGTCGTAGACAGCTCTAGCACCGCT TAAACGCACGTACGCGCTGTCCCCCGCGTTTTAACCGCCAAGGGGATTACTCCCTAGT CTCCAGGCACGTGTCAGATATATACATCCGAT
Primer-fw	5'- biotin- ATCGAGAATCCCGGTGCCGAG -3'
Primer-rev	5'- ATCGGATGTATATATCTGACACGTGCCTGGAGA -3’
ODN2[a]	
Template (5' to 3')	TTCTTGGCTGTGGCTCTGCGTCCTTGTCCTGAGGCCATCACAGCGCATGAACGACGAG GCACAACAGAGAGCAACACCGCCGAGGA
Primer-fw	TTCTTGGCTGTGGCTCTGCGTCCTTGTCCT
Primer-rev	TCCTCGGCGGTGTTGCTCTCTGTTGTGCCT
ODN3[a]	
Template (5' to 3')	GCTCGCTTTGTTGGTTTCCTTGTTCTCTGTGAGGCCATCACAGCGCATGAACGACGAA AGCAGCGCGAGCAAGCGAGACAGGACAC
Primer-fw	GCTCGCTTTGTTGGTTTCCTTGTTCTCTGT
Primer-rev	GTGTCCTGTCTCGCTTGCTCGCGCTGCTTT

a) See section 2 for preparation of ODNs containing nucleobase modifications.

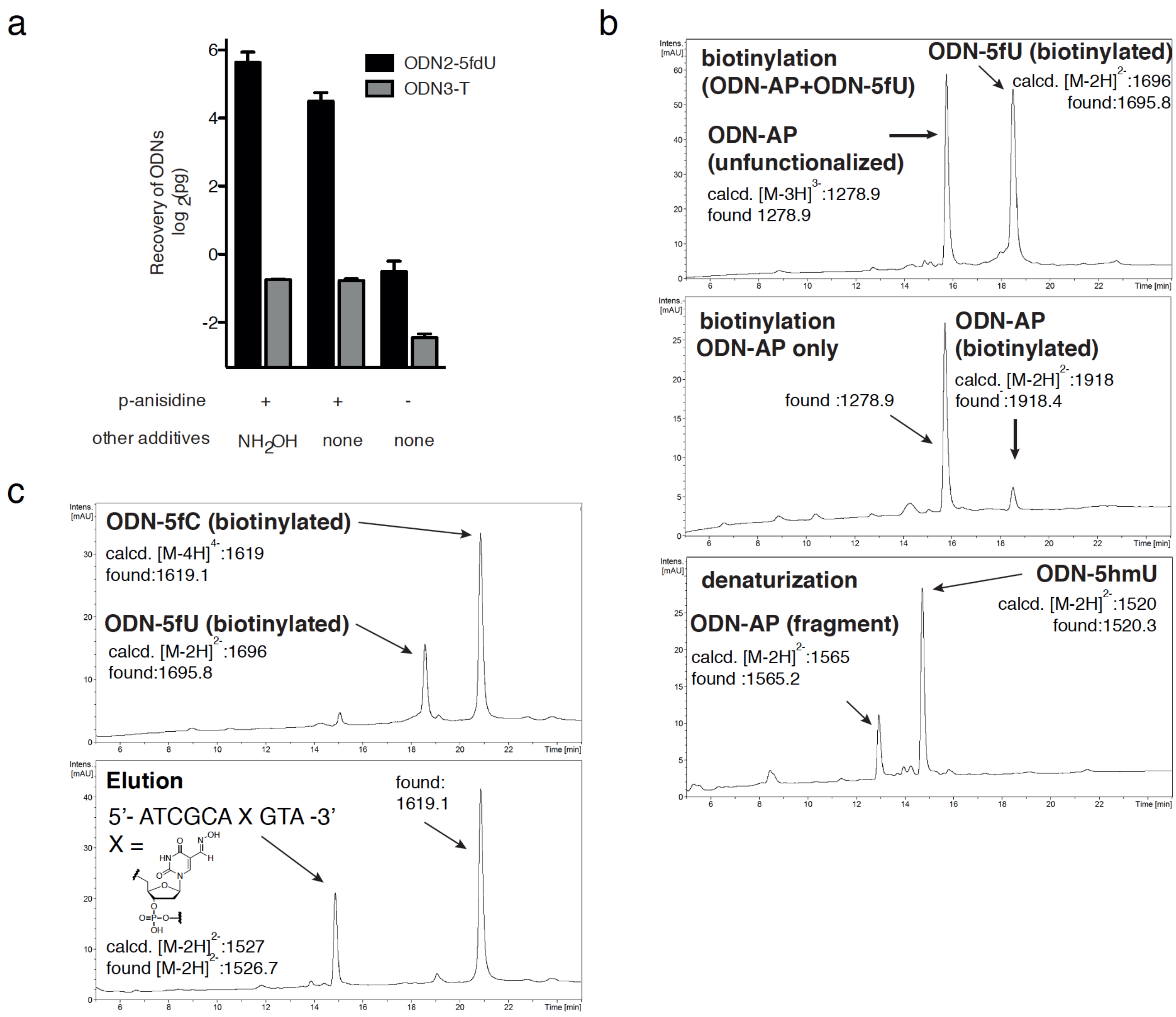

Fig. S1 | Chemical pull down of modified ODNs. (a) Recovery of 86mer ODNs (ODN2-5fU and ODN3-T) in different elution conditions. ODNs (100 pg as input) bound to the beads were incubated (40 ºC for 1 h) with 20 mM phosphate buffer (pH 6) supplemented with *p*-anisidine (5mM) and 0.05% (v/v) hydroxylamine as indicated. The recovery of ODNs was estimated by qPCR. Data set is shown as a mean±SD of three replicates. (b) A LC trace of a mixture of ODN-AP and ODN-5fU subjected to the biotinylation condition used in the 5hmU mapping *in Leishmania* samples (top). A LC trace of ODN-AP subjected to the same condition (middle). Minor biotinylated component derived from ODN-AP overlapped to the biotinylated ODN-5fU in the top panel. A LC trace of a mixture of ODN-AP and ODN-5hmU subjected to the denaturation condition (0.05 M NaOH, bottom). (c) LC-MS traces of 5fC modified ODN after biotinylation (top), followed by treatment in a 40 mM phosphate buffer (pH 7) supplemented with *p*-anisidine and 0.05% (v/v) hydrazine at 40 ºC for 1h (bottom).

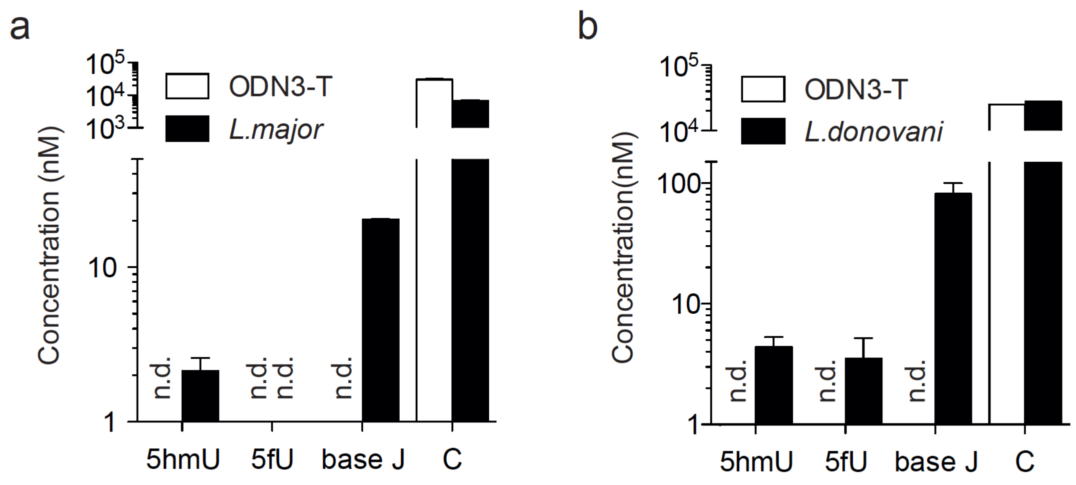

Fig. S2 | DNA nucleobase modification profiles of *L. major* (a) and *L. donovani GR383WT1* (b). Digested genomic DNA samples (0.4 µg) were analyzed by LC-MS^2. Non-modified ODN3 was used as the negative control. Data were shown as mean±SD for three technical triplicates. Lower detection limit of each modifications, which was determined by measuring synthetic mononucleoside standards, were 0.6 nM for 5hmU and 5fU, and 2.5 nM for base J (n.d.: not detected). The percentages of T modification in the DNA samples were calculated by the following equation based on the reported GC contents in the genomes: %T modification per nucleobases = 0.5*(%GC in the genome)*(the concentration of T modification)/(the concentration of C).

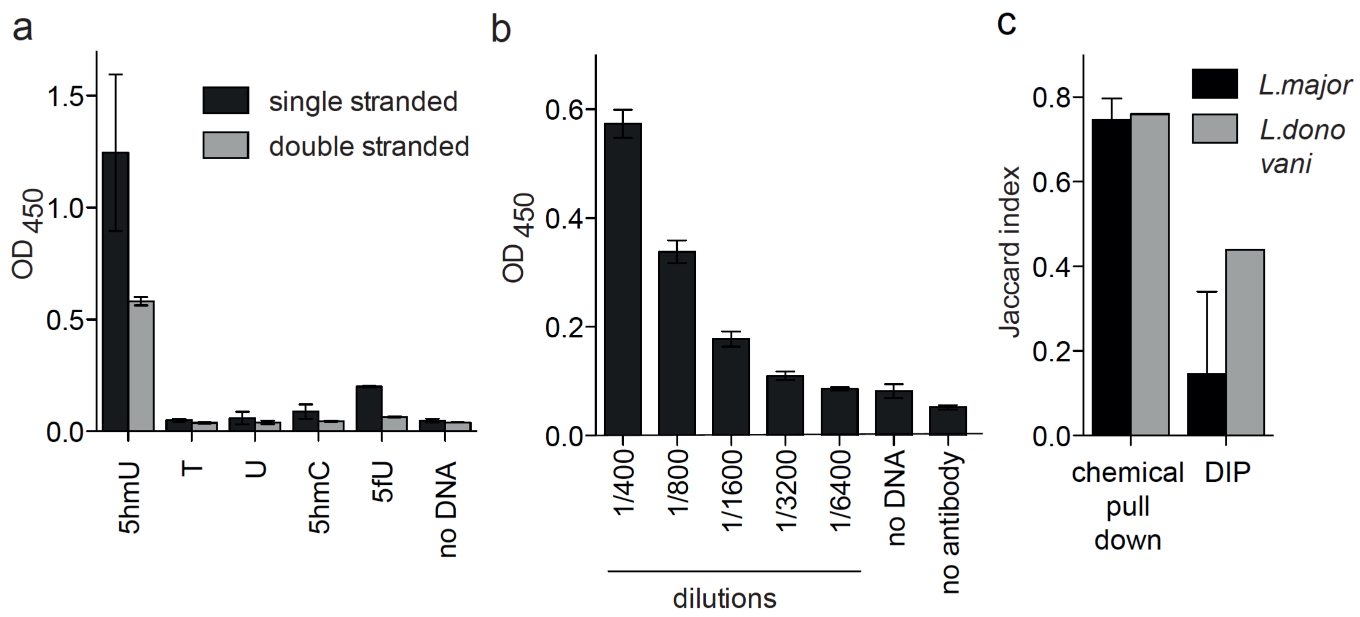

Fig. S3 | 5hmU specificity of the antibody used for 5hmU-DIP. (a) Binding of ab19735 (500 fold dilution) to ODN1 bearing various nucleobase modifications. (b) Concentration dependent binding of ab19735 to ODN1-5hmU. (c) Jaccard index showing degree of peak overlaps within technical replicates. Jaccard index is computed as the length of the intersection divided by the length of the union between the two sets of intervals (i.e. bed files). The value 1 means that peaks overlap completely, and value 0 means peaks had no overlap. Values for *L. major* were shown as mean of Jaccard indexes obtained in three combinations of two technical replicates out of three.

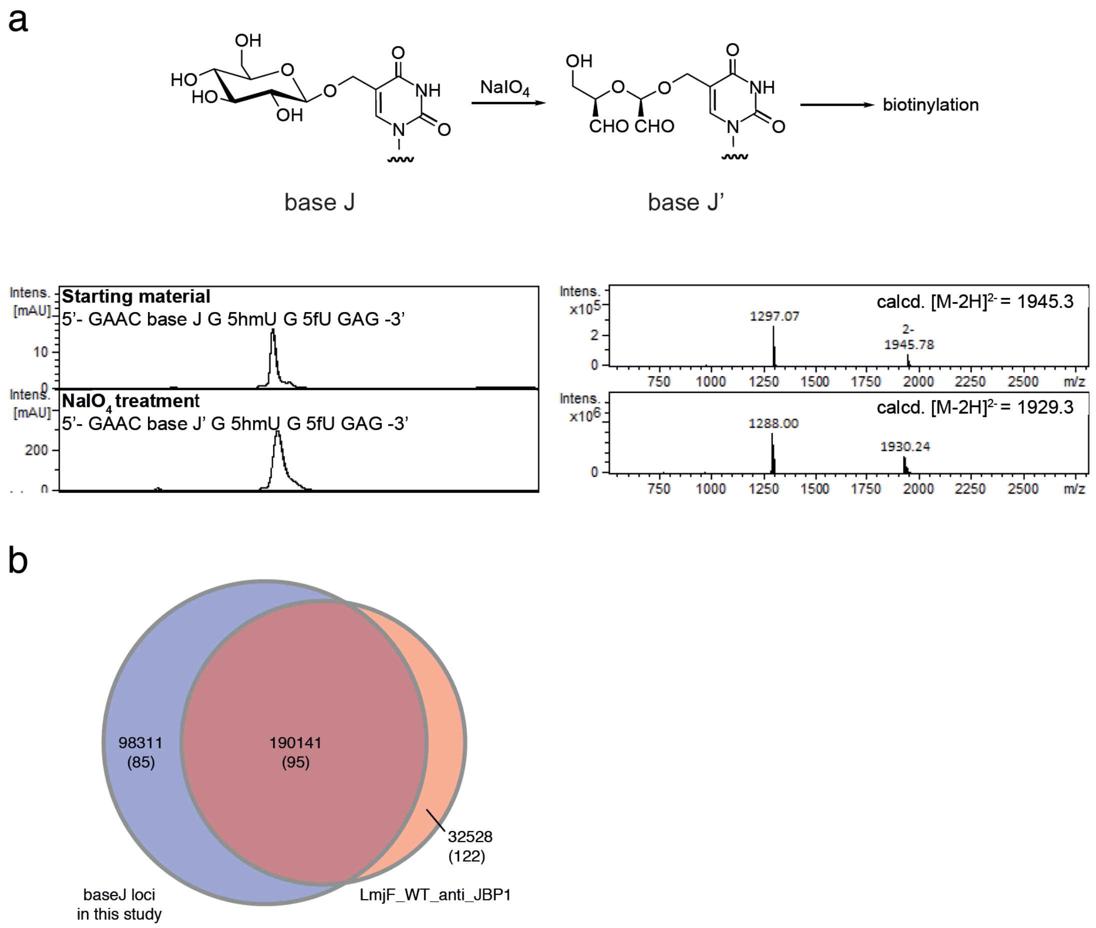

Fig. S4 | Base J chemical pull down. a) Schematic picture of base J pull down and LC trace of ODN modified base J, 5hmU and 5fU (top) and LC trace after treatment by $NaIO_4$ (bottom). ESI-MS values at the largest peak found in each sample are shown on the right panel. b) Base J loci consistency between the chemical enrichment method and previously reported JBP-affinity based enrichment (van Luenen et al.). [2] Overlaps of base J loci are shown as total length of peaks (bp) and peak numbers (in brackets).

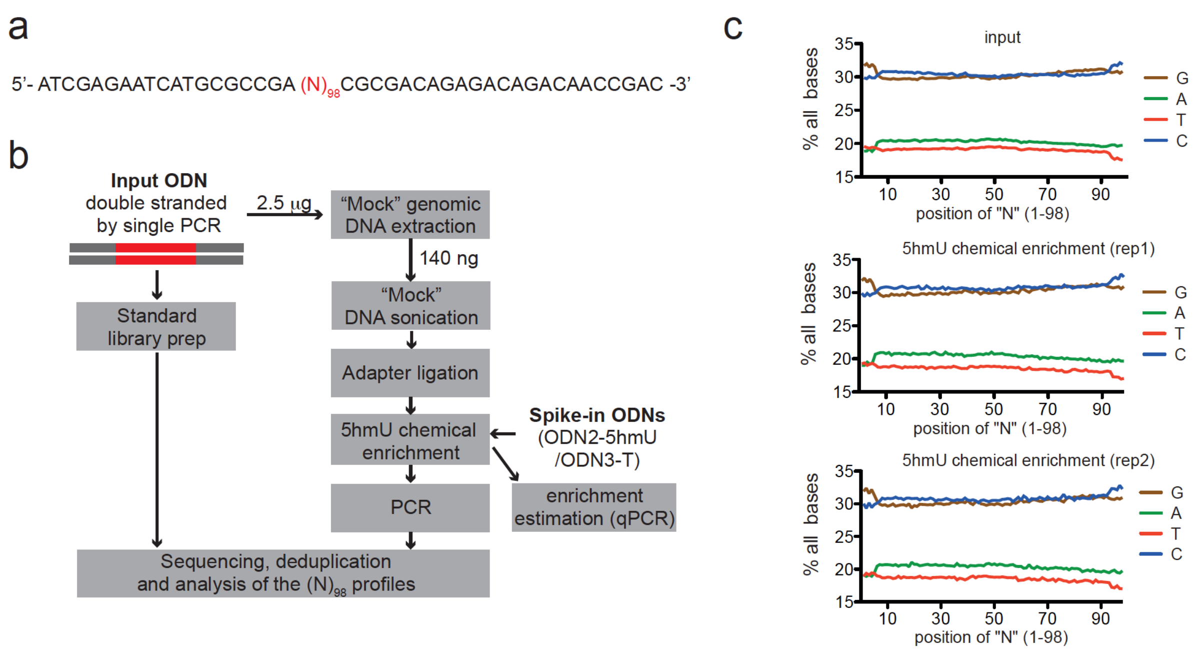

Fig. S5 | Assessment of experimental biases. To assess potential artifacts and biases in the chemical 5hmU enrichment process, we carried out a model study using chemically synthesized randomized ODNs. We considered that potential artifacts may arise from the oxidative damage to thymine (to either 5hmU or 5fU) in any process before enrichment (genomic DNA extraction, DNA fragmentation, adapter ligation, oxidation, and biotinylation), and also that the sequence bias may potentially arise from PCR or background binding of DNA to the streptavidin beads. The model ODNs do not bear 5hmU and 5fU, therefore are suitable to study potential oxidative damage during DNA treatment and background artifact signals. (a) The chemically synthesized model ODN sequence, where N means a randomly incorporated A, T, G or C. The average GC content of the randomized region was adjusted to 64% during the template DNA synthesis (equal to the GC content of L. major genome). (b) The experimental workflow. The double-stranded input DNA was subjected to either standard library prep or "mock" DNA extraction, where the input DNA purified by a genomic DNA extraction kit using DNeasy Blood & Tissue Kit (Quagen, followed protocol provided for cultured cells) for cultured cell samples. The extracted DNA was then subjected to sonication using the same protocol used to fragment L. major DNA ("mock" fragmentation). Although the input DNA was naked DNA and also too short to be further fragmented by sonication, it was necessary to carry out the two "mock" processes in order to assess oxidative damages during the actual workflow. The DNA was then adapter ligated, oxidized, and biotinylated, followed by enrichment. To exclude the possibility that enrichment was not successful, two spike-in controls were added to the library after adapter ligation and used to estimate the 5hmU enrichment. After sequencing, duplicated reads that arose due to PCR biases were removed, and sequence profiles in the randomized region were analyzed by fastqc. (c) ATGC contents of the randomized regions in input (left) and 5hmU pull down samples (middle and right). We did not observe any increase of AT contents in 5hmU pull down

samples, which would have been observed in case of oxidative damage during DNA treatment. We also did not observe any specific sequence motif (in five bases context) enriched in 5hmU pull down samples, suggesting our 5hmU enrichment procedure does not induce any noticeable sequence bias.

Table S3 | Libraries generated in this study.

Species	Method	Reads	Alignment	Enrich Ment[e]	Filenames
L. major[a]	DIP (5hmU)	6890424	9317959	87	fk041_Lmaj_DIP1_S2_L001_R1_001.fq.gz<br>fk041_Lmaj_DIP1_S2_L001_R2_001.fq.gz
L. major[a]	DIP (5hmU)	8162807	11831752	31	fk043_Lmaj_DIP2_S3_L001_R1_001.fq.gz<br>fk043_Lmaj_DIP2_S3_L001_R2_001.fq.gz
L. major[a]	no-oxidation ctrl [d]	2550858	2294020	105	fk045_Lmaj_nooxctrl1_S1_L001_R1_001.fq.gz
L. major[a]	no-oxidation ctrl[d]	2961421	1851450	186	fk046_Lmaj_nooxctrl2_S2_L001_R1_001.fq.gz
L. donovani	DIP (5hmU)	3060900	2514914	25	fk047_Ldon_DIP1_S6_L001_R1_001.fq.gz
L. donovani	DIP (5hmU)	3991278	3212455	52	fk048_Ldon_DIP2_S7_L001_R1_001.fq.gz
L. major[a]	Chem (5hmU)	3184545	2224973	75	fk050_Lmaj_chem1_S3_L001_R1_001.fq.gz
L. major[a]	Chem (5hmU)	5336350	3703347	98	fk051_Lmaj_chem2_S4_L001_R1_001.fq.gz
L. major[b]	DIP (5hmU)	4634103	2853102	- [f]	fk052_Lmaj_DIP3_S1_L001_R1_001.fq.gz
L. major[b]	Chem (5hmU)	4278364	3261394	143	fk054_Lmaj_chem3_S3_L001_R1_001.fq.gz
L. major[b]	Input	2289079	1836039	1	fk059_Lmaj_input_S8_L001_R1_001.fq.gz
L. donovani	Chem (5hmU)	1533974	1196975	16	fk066_Ldon_chem1_S1_L001_R1_001.fq.gz
L. donovani	Chem (5hmU)	1668973	1272443	83	fk067_Ldon_chem2_S2_L001_R1_001.fq.gz
L. donovani	no-oxidation ctrl[d]	1443814	1137891	-[f]	fk068_Ldon_nooxctrl1_S4_L001_R1_001.fq.gz
L. donovani	no-oxidation ctrl[d]	2105672	1571818	-[f]	fk069_Ldon_nooxctrl2_S5_L001_R1_001.fq.gz
L. donovani	Input	1424986	1168107	1	fk070_Ldon_input_S3_L001_R1_001.fq.gz
L. major[c]	Total RNA	8028595	1877731	-[f]	fk094_Lmaj_totalrna1_S1_L001_R1_001.fq.gz<br>fk094_Lmaj_totalrna1_S1_L001_R2_001.fq.gz
L. major[c]	Total RNA	9645786	1582510	-[f]	fk095_Lmaj_totalrna2_S2_L001_R1_001.fq.gz<br>fk095_Lmaj_totalrna2_S2_L001_R2_001.fq.gz
L. major[c]	Total RNA	7025678	1649894	-[f]	fk096_Lmaj_totalrna3_S3_L001_R1_001.fq.gz<br>fk096_Lmaj_totalrna3_S3_L001_R2_001.fq.gz
L. major[b]	Chem (base J)	3408837	2849893	-[f]	fk113_Lmaj_baseJ1_S3_L001_R1_001.fq.gz
L. major[b]	Chem (base J)	2930727	2500030	-[f]	fk116_Lmaj_baseJ2_S4_L001_R1_001.fq.gz
L. major[c]	Chem (base J)	4057103	3432863	-[f]	fk117_Lmaj_baseJ3_S5_L001_R1_001.fq.gz

a) Lot 60685413 was used; b) lot 63717803 was used; c) lot ATCC-CUST-30012D was used; d) libraries were prepared with chemical pull down without oxidation ("no-oxidation" controls); e) enrichments were estimated by read counts for each spike-in controls (ODN2-5hmU/ODN3-T or ODN2-T/ODN3-5hmU for 5hmU enriched libraries, ODN2-5fU/ODN3-T for "no-oxidation" control libraries) followed by normalization by the values of no pull down controls; f) spiked-in ODNs were not used.

Table S4 | Motif sequences obtained by DREME analysis

Motifs	E-values[a]
**5hmU/base J loci**	
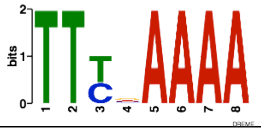	3.7e-037
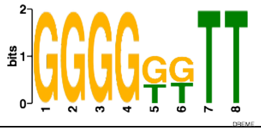	2.4e-035
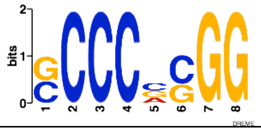	2.3e-029
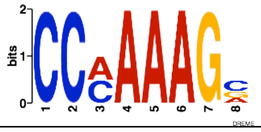	1.3e-028
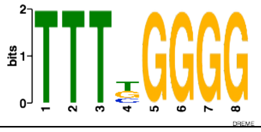	1.9e-024
**5hmU only loci**	
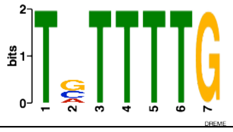	5.9e-003
**Base J only loci**	
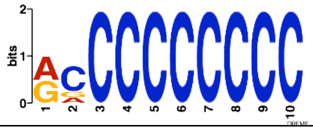	1.5e-006
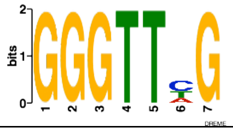	4.9e-006
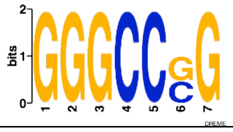	2.4e-005
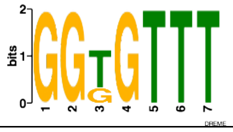	2.7e-005
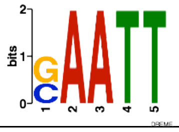	2.7e-003

a) The motif p-value times the number of candidate motifs tested.

2. Supplemental Procedures

2-1. General information

General reagents and equipment

Ultrapure water produced by Synergy® UV Remote Water Purification System (Merck Millipore) or PCR grade water (Roche Life Science) was used to prepare all aqueous solutions unless specified. DNA LoBind Tubes (Eppendorf) were used throughout the procedure. Incubation and PCR was carried out using T100 Thermal Cycler (BioRad). qPCR experiments were carried out with CFX96 Real-Time System (BioRad). Sonication of DNA was carried out with M220 Focused-ultrasonicator™ (Covaris). All sequencing experiments were carried out on a Mi-Seq using Miseq reagent kit v3 (Illumina).

Buffer compositions used in the study

Binding buffer 1 (x1): 10 mM Tris (pH 7.5), 1 mM EDTA, 2M NaCl, 0.1% (v/v) tween 20

Elution buffer: 0.05% (v/v) hydroxylamine (Sigma Aldrich), 5 mM anisidine (Sigma Aldrich), 0.1% (v/v) tween 20 in 50 mM phosphate buffer (pH 6)

Binding buffer 2: 0.1% (v/v) tween 20 in PBS (x1)

ELISA buffer (x1): 50 mM K_2HPO_4 (pH 7.4), 100 mM KCl and 3% (w/v) BSA

2-2. Preparation of DNA models listed in Table 2

Biotinylated ODNs bearing 5hmU, U, 5fU, 5mC, 5hmC as well as a control ODNs without any nucleobase modification were synthesized by PCR. Each ODN template (10 nM) was amplified in the presence of indicated primers (1 μM each) and dNTPs (0.2 mM each) using Dreamtaq DNA polymerase (1.25 unit, Life Technologies). 5hmUTP, 5fUTP, or dUTP were used instead of TTP for 5hmU, 5fU, U modified ODNs. 5hmdCTP and 5mdCTP was used instead of dCTP for 5hmC, 5mC modified ODNs. Incorporation of each nucleobase modification to ODNs was confirmed by HPLC.

2-3. Reactions with oligo DNA models

To solution of ODNs (10 μM) in 50 mM NaOH (24 μL), a 10-fold diluted oxidant solution (1 μL) provided in the TrueMethyl™ kit (Cambridge Epigenetics) was added and the resulting mixture was incubated at rt for 30 min. After the incubation, the mixture was passed through mini quick spin oligo columns (Roche Life Science). The oxidised DNA solution (ca. 25 μL), 100 mM phosphate buffer (pH 6, 10 μL), 17 mM (+)-biotinamidohexanoic acid

hydrazide (15 μL, Sigma Aldrich) were mixed and incubated at 40 °C for 2 h. The reaction mixture was passed through mini quick spin oligo columns (Roche Life Science) or Micro Bio-Spin columns with Bio-Gel P-6 in SSC Buffer (P6 column, Bio Rad), which were pre-washed with water three times prior to use. The biotinylated DNA solution was mixed with the same amount of elution buffers (x 2) as indicated in Fig. 1c and incubated at 40 °C for 1h. The reaction mixture was passed through a mini quick spin oligo column or P6 column, which was pre-washed with water three times prior to use.

LC-MS analysis of oligo DNA models

LC-ESI MS spectra were recorded on an AmaZon X ESI-MS (Bruker) connected to an Ultimate 3000 HPLC (Dionex). Analyses of ODNs were carried out by LC-MS using XBridge BEH C18 XP (30Å, 2.5 μm, 3 mm x 50 mm, Waters) or XTerra MS C18 Column (125Å, 2.5 μm, 2.1 mm x 50 mm, Waters). The elution solvents were 10 mM triethylamine and 100 mM hexafluoroisopropanol in water (solvent A) and methanol (solvent B). The flow rate was 0.2 ml/min. After equilibration at 5% B, a liner gradient of 2 min at 5% B, 20 min at 5–30% B, 3 min at 30% B was used as the elution condition.

2-4. qPCR analysis to assess enrichment efficacy of chemical pull down (relevant to Fig. S1)

A solution (25 μL) of ODN2-5fU, ODN3-T (100 pg for each ODNs) and salmon sperm DNA (1μg, Life Technologies) was subjected to the biotinylation as described above, followed by enrichment as described for the Chemical 5hmU enrichment sequencing. The recovered ODN2-5fU and ODN3-T were quantified with Brilliant III Ultra-Fast SYBR Green qPCR Master Mix (5 μL) (Life Technologies). Enrichment was calculated as (recovery of ODN2-5fU)/(recovery of ODN3-T) assessed by qPCR quantification

2-5. Validation of 5hmU specificity of ab19735 by ELISA (relevant to Fig. S3)

Streptavidin coated high sensitivity plates (Life Technologies) were washed with ELISA buffer (3 x 200 uL, see section 2-1 for the buffer composition) and incubated with 5'-biotinylated ODN1 (10 ng/μL, 50 μL per well) at 4 °C for overnight, followed by washing 3 times with ELISA buffer (200 uL). The plate was incubated with urea (5 M in water) at 40 °C for 30 min, followed by quick washing 3 times with ELISA buffer (200 uL). Experiments using double-stranded DNAs were carried out without this urea treatment step. The plate was pre-blocked with ELISA buffer (200 uL) at room temperature for 3 h, followed by washing 3 times with ELISA buffer (200 uL). The plate was incubated with antibody ab19735 (Abcam, RRID:AB_722498) at rt for 1 h in dilutions indicated in Fig.

S3ab, followed by wash 3 times with ELISA buffer (200 uL). The plate was then incubated with protein G-HRP (1:5,000 dilution, Life Technologies) at rt for 1 h in dilutions indicated in Fig. S4ab, followed by washing 3 times with ELISA buffer (200 uL). The bound protein G-HRP was detected by addition of the substrate BM Blue POD Substrate (Roche Life Science) followed by the addition of 1M H_2SO_4 (50 uL). The absorbance at 450 nm was measured on a a SPECTROStar Nano microplate reader (BMG LABTECH).

2-6. Mononucleoside composition analysis by LC-MS^2 (relevant to Fig. S2)

Synthesis of stable isotope labelled mononucleosides

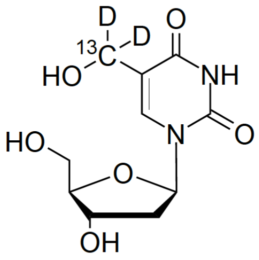

1

(5-[$^{13}CD_2$] hydroxymethyl-2'-deoxyuridine 1) To a solution of 2-deoxyuridine (173 mg, 0.75 mmol) in ^{13}C-d_2-formaldehyde (1.5 mL, 20% by weight in D_2O) under an argon atmosphere was added NEt_3 (1.35 mL, 9.70 mmol). The reaction was sealed and heated for 72 h at 60 °C. The reaction mixture was concentrated and purified by flash column chromatography (CH_2Cl_2:MeOH = 6:1 to 5:1, v/v) to afford **1** as a white solid. (72 mg, 37%); ^{1}H NMR (500 MHz, d4-methanol) δ 7.98 (d, $J_{13C\text{–}H}$= 4.1 Hz, H6), 6.32 (app t, J = 6.8 Hz, H1'), 4.42 (dt, J = 6.5 Hz, 3.4 Hz, H3'), 3.94 (app q, J = 3.4 Hz, H4') 3.81 (dd, J = 12.0 Hz, 3.4 Hz, H5'), 3.75 (dd, J = 12.0 Hz, 3.9 Hz, H5') 2.26 (m, 2H, H2'). ^{13}C NMR (126 MHz, MeOD) δ 161.5 (C4) 152.2 (C2), 139.5 (d, $J_{13C\text{–}13C}$ = 4.6Hz, C6), 115.3 (d, $J_{13C\text{–}13C}$ = 53.0 Hz, C5), 88.9 (C4'), 86.5 (C1'), 72.3 (C3'), 62.9 (C5'), 57.4 (q, $J_{13C\text{–}D}$ = 21.4 Hz, hydroxymethyl), 41.3 (C2'); HRMS $C_9{}^{13}CH_{13}D_2N_2O_6$ $[M+H]^+$ calcd. 262.1089, found 262.1096.

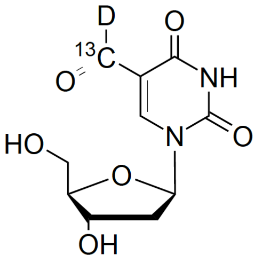

2

(5-[^{13}CD] formyl-2'-deoxyuridine 2) To a solution of **2** (40 mg, 0.15 mmol) in MeOH (1 mL) was added MnO_2 (67 mg, 1.25 mmol) and the reaction mixture was stirred at 50 °C for 18 h. The catalyst was removed by filtering through celite, and the filtrate was concentrated. The crude product was purified by flash column chromatography (CH_2Cl_2:MeOH = 9:1 to 5:1, v/v) to afford **2** as a white solid (3.1 mg, 8%); ^{1}H NMR (500 MHz, D_2O) δ 8.68 (d, $J_{13C\text{–}H}$ = 4.8 Hz, 1H, H6), 6.14 (app t, J = 6.5 Hz, 1H, H1'), 4.37 (dt, J = 6.5, 4.7 Hz, 1H, H3'), 4.01 (td, J = 4.7, 3.3 Hz, 1H, H4'), 3.79 (dd, J = 12.6, 3.3 Hz, 1H, H5'), 3.68 (dd, J = 12.6, 4.7 Hz, 1H, H5'), 2.43 (m, 1H, H2'), 2.33 (m, 1H, H2'). ^{13}C NMR (126 MHz, D_2O) δ 188.7 (t, $J_{13C\text{–}D}$= 27.3 Hz, formyl), 163.0 (C6), 151.2 (d, J13C-13C = 6.4 Hz, C4), 150.4 (C2), 111.2 (d, $J_{13C\text{–}13C}$ = 61.4 Hz, C5), 87.2 (C1'), 86.9 (C4'), 69.8 (C3'), 60.6 (C5'), 39.6 (C2'). HRMS $C_9{}^{13}CH_{11}DN_2NaO_6$ $[M+Na]^+$ calcd. 281.0689, found 281.0692.

LC-MS²

The genomic DNA sample (0.4 μg) was treated with DNA degradase™ (0.1 U/μL, ZYMO RESEARCH) in the presence of 5hmU-SI (20 nM), 5fU-SI (20 nM), and deanimase inhibitors erythro-9-amino-β-hexyl--α-methyl-9H-purine-9-ethanol hydrochloride (10 nM, Cayman Chemical) and 3,4,5,6-tetrahydrouridine (10 nM, Calbiochem). After incubation at 37 ºC for 4 h, the solution was filtered with Amicon Ultra-0.5 mL 10 K centrifugal filters (Merck Millipore) and subjected to LC-MS² analysis on Q Exactive™ Hybrid Quadrupole-Orbitrap Mass Spectrometer (Thermo Scientific) equipped with a nano spray ionization source, coupled to an Ultimate RSLCnano LC system (Dionex). A capillary column (hypercarb, 5 cm x 75um ID, 3 um particle size, self packed) was used and water - acetonitrile containing 0.1% formic acid was the solvent system with 1.5 μL/min flow rate, and gradient of 2% (0–5 minutes); 2–40% (5–12 minutes); 40–95% (12–15) acetonitrile by a 3 minute equilibration step. The quadrupole was pre-set to isolate the precursor-ions of all analytes ($[M+H]^+$ ±2 m/z). Settings were as follows: spray voltage: 2300 V, capillary temperature: 250 °C, resolution 35.000, NCE: 10.0, AGC target: $1e^6$, 100 ms maximum injection time. For the quantitation, calibration curves were generated by recording data for a dilution series of the analytes (C 10000–30 nM, 5hmU 20000–0.63 nM, 5fU 1000–0.63 nM and base J 2000–2.5 nM) using stable isotope labeled $^{15}N_3C$, $^{13}CD_2$5hmU and ^{13}CD5fU as internal standards at a 20 nM concentration. Base J was quantitated using external calibration of the calibration line. The limits of quantitation were 0.37 nM, 0.67 nM and 1.25 nM for 5hmU, 5fU and base J respectively. All data was processed using the Thermo Xcalibur quan browser software.

2-7. 5hmU-DIP (relevant to Fig. S3)

Fragmented DNA samples (200 ng) and a solution of spike-in control ODNs (100 pg for each, see Table S2) were mixed and used for adapter ligation using the standard protocol and reagents provided in NEBNext® Ultra™ DNA Library Prep Kit for Illumina® (New England BioLabs) and sequence adapters provided in TruSeq DNA Sample Preparation Kit v2 (Illumina). The adapter ligated DNA samples were purified by AMPure® XP Beads (Beckman Coulter) and eluted with 25 μL of water. For generating 5hmU-enriched library by DIP, the adapter ligated DNA (20 μL) was heated to 95 °C for 10 min and then immediately cooled on ice to denature. To the solution, binding buffer 2 (2x, 35 μL, see section 2-1 for the buffer composition), anti-5-hydroxymethyluridine antibody ab19735 (5 μL, Abcam, RRID:AB_722498), and rabbit anti-goat IgG H&L ab6697 (10 μL, Abcam, RRID:AB_955988) were added. The mixture was incubated with rotation at 4 °C overnight. The solution was added to Dynabeads® Protein G (Life Technologies) which had been pre-washed with citrate‒phosphate buffer (pH 5.5) and binding buffer (x1,

200 μL). The supernatant was removed, and the beads were washed 3 times with binding buffer 2 (1x, 200 μL). The beads were re-suspended in an elution buffer (50 mM Tris-HCl pH 8, 10 mM EDTA, and 0.5% SDS) and treated with proteinase K (20 μg/μL, 1.75 μL). The mixture was incubated at 50 °C and 1300 rpm for 2 h and placed on a magnetic rack. The supernatant containing the eluted DNA was collected, and purified using the GeneJet PCR purification kit. The obtained 5hmU enriched libraries and the control libraries were amplified by amplified by PCR Master Mix and PCR Primer Cocktail provided in TruSeq DNA Sample Preparation Kit v2 (Illumina) and quantified by KAPA Library Quantification Kit (KAPA Biosystems).

2-8. UV melting temperature analysis (relevant to Fig. 3a)

ODN with a sequence of 5'-GCCCXXXXXXXXGGCG-3' (X = 5hmU or T, 5 μM, Eurogentec) was annealed with its complementary ODN with a sequence of 5'- CGCCAAAAAAAAGGGC-3' (5 μM, Eurogentec) in PBS buffer. Samples in quartz cuvettes with 1 cm path length were heated from 10 °C to 90 °C at the ramping rate of 1 °C /min, then cooled from 90 °C 10 °C at the rate of 4 °C/min. The heating-cooling cycle was repeated for three times, with data collected at every 1 °C, and UV absorbance at 260 nm was recorded with Cary 100 UV-Vis Spectrophotometer (Agilent). Tm values were defined as the temperature where half of the duplex is dissociated in the heating process.

2-9. Strand sensitive total RNA-seq (relevant to Fig. 3b)

Total RNA (1 μg) was fragmented by incubating in a fragmentation buffer (40 mM Tris-HCl pH 8.2, 100 mMLiCl, and 30 mM $MgCl_2$) followed by purification using RNA Clean & Concentrator 5 (Zymo Research). The RNA was treated in T4 PNK (Thermo Fisher Scientific) in absence of ATP, and purified using RNA Clean & Concentrator 5. The adapter (5'- 5rApp AGATCGGAAGAGCACACGTCTG-SpC3- 3') was ligated to the RNA by T4 RNA ligase 2 K227Q (New England BioLabs) then purified using RNA Clean & Concentrator 5. Reverse transcription was carried out using the reverse primer (5'-CAGACGTGTGCTCTTCCGATCT-3') and SuperScript® III Reverse Transcriptase (Thermo Fisher Scientific) followed by RNA degradation by heating up samples at 95 °C in presence of NaOH (0.1 mM final concentration) and purification by geneJET PCR purification kit (Thermo Fisher Scientific). ssDNA adapter was ligated to the resulted single-stranded DNA, by following literature protocols. [3] The obtained samples were amplified by NEBNext® High-Fidelity 2X PCR Master Mix (New England BioLabs) and primers (5'-CAAGCAGAAGACGGCATACGAGATNNNNNNGTGACTGGAGTTCAGACGTGTGCTCTTCCGATC-s-T-3'

and 5‘-AATGATACGGCGACCACCGAGATCTACACTCTTTCCCTACACGACGCTCTTCCGATC-s-T-3‘). The libraries were quantified using KAPA Library Quantification Kit (KAPA Biosystems) by following the standard protocol provided with the kit and sequenced by Illumina MiSeq with paired-end read length 75bp x2.

2-10. Assessment of experimental biases using a randomised ODN (relevant to Fig. S5)

The double-stranded randomized ODN (Fig. S5a) was prepared by single extension of the ODN template purchased from biomers.net. The primer 5’-GTCGGTTGTCTGTCTCTGTCGCG-3’ (Thermo Scientific) and the template in NEB2 buffer (NEB) were annealed at 95 º C for 3 min. Then dNTP mix and Klenow Fragment (3'-->5' exo-) (NEB) was added at 37 º C followed by 1 h incubation. The double stranded ODN was purified by geneJET PCR purification kit. The libraries were prepared as described in the 5hmU chemical enrichment procedures. The procedures for sequence data analysis are available from the github repository (https://github.com/sblab-bioinformatics/mapping-5hmU-in-Leishmania).

1. Kawasaki F, Murat, P., Li, Z., Santner, T., Balasubramanian, S. Synthesis and biophysical analysis of modified thymine-containing DNA oligonucleotides. *Chem. Commun.* 2017; DOI:10.1039/c6cc08670e.
2. van Luenen HG, Farris C, Jan S, Genest PA, Tripathi P, Velds A, Kerkhoven RM, Nieuwland M, Haydock A, Ramasamy G, et al. Glucosylated hydroxymethyluracil, DNA base J, prevents transcriptional readthrough in Leishmania. *Cell* 2012;150:909-921.
3. Kwok CK, Balasubramanian S. Targeted Detection of G-Quadruplexes in Cellular RNAs. *Angew. Chem. Int. Ed.* 2015;54:6751-6754.
